# Trust Me I'm a Doctor; the Importance of Trust in Promoting High Performance Learning in Medical Education

**DOI:** 10.15694/mep.2020.000184.1

**Published:** 2020-08-27

**Authors:** Annwyne Houldsworth

**Affiliations:** 1 Khalifa University

**Keywords:** Trust, educational leadership, teaching and learning, professionalism, educational neuroscience, self authorship, mentoring and coaching

## Abstract

This article was migrated. The article was not marked as recommended.

Trust is an essential component of developing bonds and, in particular, the relationship between teacher and learner. The cumulative effects of pedagogy, curriculum, content, and delivery in teaching and learning of medical students (MS) are well established and the importance of the relationship between student and teachers, with particular reference to the concept of trust is reviewed, addressing aspects of intention, capability, character, and integrity.

Trust is often perceived as a soft quality with respect to education, however trust actually provides an environment of hope and inspirational optimism. In such an environment teachers and learners can be authentic about their ‘best selves’, developing good character with high emotional intelligence (EI), where honest reflection is the key to enhanced integrity with transparent intentions in their relationships.

Trusting relationships in education instil mutual respect, enhance collaboration, and promote the independent thinking that results from transparent and kind mutual interactions. Indeed, loyalty and commitment to values and goals ensures the success of the learning environment.

Neuroscience and psychology experiments demonstrate recent evidence to support the importance of trust in relationships that can be considered relevant to teaching and learning. The expression of hormones and brain function, associated with trusting relationships and interpersonal bonding is explored.

## Introduction

*“When the trust account is high, communication is easy, instant, and effective.”* -Stephen R. Covey

Fides is the mythological Roman Goddess of trust, a religious deity with a symbolic narrative, based on an important virtue required for honour and credibility in all contractual arrangements, including marriage. She oversaw the moral integrity of the Romans. From the name of this goddess we derive the word fidelity as a quality of faithfulness or loyalty. The Latin *fidere* means ‘to trust’. Pistis was the Greek equivalent, a spirit associate of truth, trust, honesty and good faith and was a good spirit or personified concept that escaped from Pandora’s box (Thurston, 1898; mythencyclopedia.com).

*“The glue that holds all relationships together-including the relationship between the leader and the led-is trust, and trust is based on integrity.”* -Brian Tracy

The concept of trust exceeds the nature of a relationship of alliance and is described as the glue that holds together partnerships. In medicine there are therapeutic alliances between clinician and patient; in law, strategic alliances (agreement between two parties) all of which benefit from the experience of mutual trust (Covey, 2006). Abu Bakr Muhammad ibn Zakariya Al Razi, the freethinking Islamic clinician (936-1013) emphasized the importance of developing trust between patient and doctor ( Lakhtakia, 2014).

**Figure 1.**
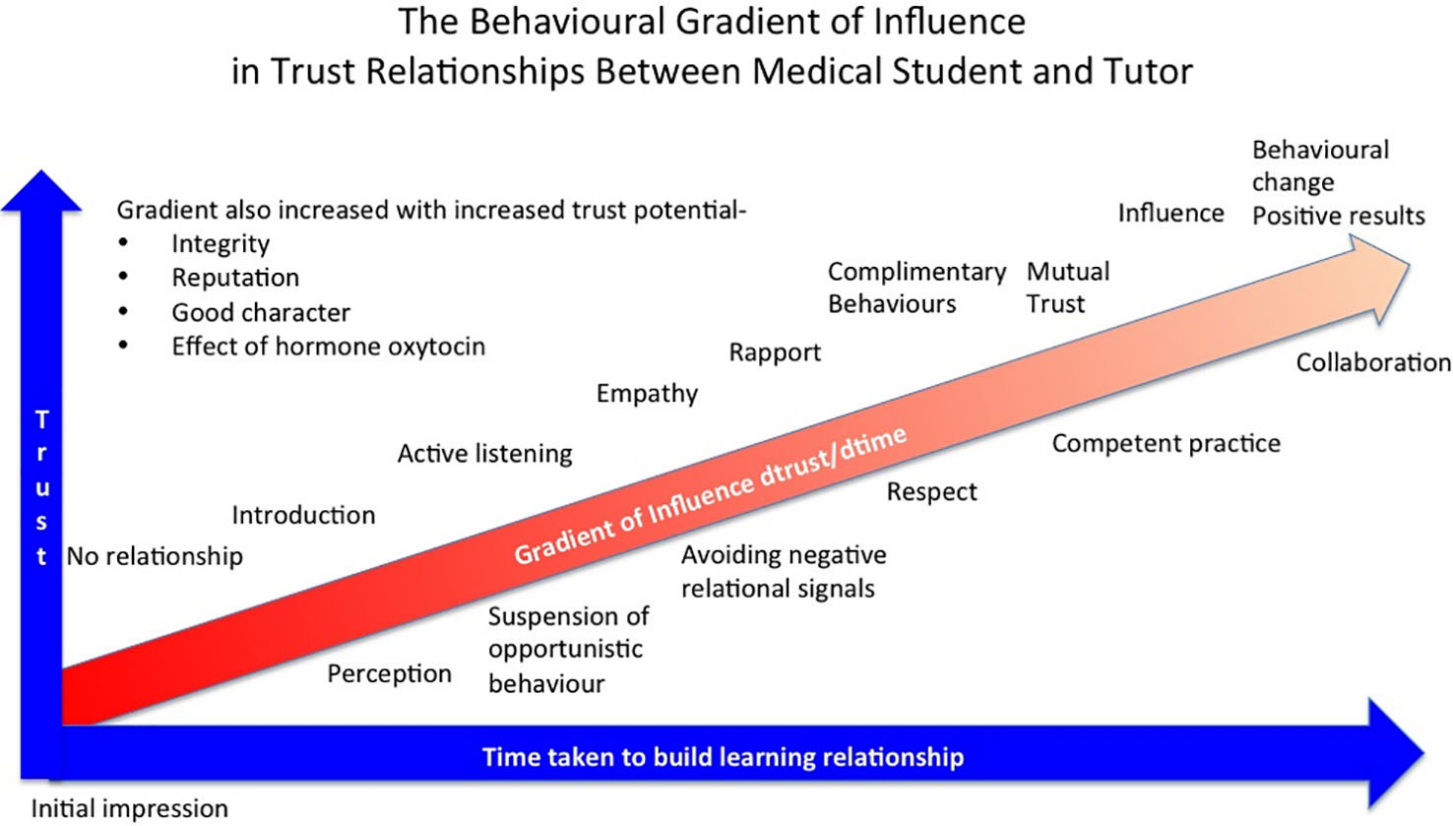
Behavioural Gradient

Trusting teacher learner relationships (TLRs) in education instil mutual respect, enhance collaboration, and promote the independent thinking that results from transparent and kind mutual interactions, also essential skills for the clinician. Indeed, loyalty and commitment to values and goals ensures the success of the learning environment. Many factors enhance trust in the learning environment, adding greater potential to the learning and speed to development ( Figure 1).

Figure 1. Describing factors that influence the development of trust in a relationship between tutor and medical student. Based on The Behavioural Stairway Influence Mode ( Ireland and Vecchi, 2009).

Evidence is building in medical education to support the power of mentoring and coaching as a method to improve medical training and education, where high levels of trust are required ( Lovell, 2018).

Often perceived as a soft quality, with respect to education, trust actually provides an environment of hope and inspirational optimism. In such an environment teachers and learners can be authentic about their ‘best selves’, developing good character, where honest reflection is the key to enhanced integrity with transparent intentions in their relationships ( Wilt, Thomas and McAdams, 2019).

Trust is an essential component of the relationship between teacher and learner, particularly as an academic tutor. The cumulative effects of pedagogy, curriculum, content, and delivery in teaching and learning of medical students (MS) are well established and the importance of the relationship between student and learners with particular reference to the concept of trust is explored in this paper ( Keiler, 2018).

## Honest reflection and evaluation

A recent study by Bellucci has observed that individuals prefer trusting honest others when required to share honest information. An honest reputation influences learning processes for trust-based interactions in social learning social interactions and honesty is central to trust and trustworthiness ( Bellucci and Park, 2020). Albert S Humphrey is credited with first developing the strengths, weaknesses, opportunities and threats (SWOT) analysis, a strategic planning method to improve performance, for evaluation purposes ( Businessball.com).

More recently, it is preferred to for participants to replace the word ‘weakness’ with ‘activities that could be improved’, ‘performed better’ or referred to as ‘limitations’. When there is trust between medical educator and medical student, individual personal reflection needs to be honest, in such a way, that it enables the realistic identification of strengths for both student and mentor to build upon and recognises areas of improvement by self and mutual analysis of tasks, actions, behaviours, attitudes and results. The integrity of this process enables inspired progression and optimism with a significant identification of improvements to be made in future practice. Best practice is affirmed and empowered and below par practice identified and improved. Evaluation, in a trusting environment, is a powerful tool to develop and ensure best practice in a supportive and empowering ethos. When this is not the case and without trust, the evaluation process can be accusing, discriminatory and disempowering ( Covey, 2008).

The Johari Window is an example of a useful model for reflection that facilitates the understanding of the relationship between themselves and others, as part of the toolkit for professional development, both for student and lecturers ( Verklan, 2007).

It is important that evaluation takes place in a ‘safe place’. The word *amanah (*أمانة) in Arabic means trust from the root word, *amina*, which means state of peace, safety and security. This describes well a safe learning environment for a medical student with their tutor (Islamic Dictionary).

## Character

*Integrity is doing the right thing even when no one is watching.* CS Lewis

Boundaries of academic integrity are not well defined in the collegiate practice of university communities ( McCabe, 2009). The obvious existing rules in academic integrity and ethics, involve absolute transparency, refraining from cheating by falsifying data, plagiarism, and by always disclosing conflicts of interest (McCabe, 2018).

Lecturers with different character traits have expressed dualistic attitudes to dealing with ethical student problems between being objective, by following strictly to the university rules or subjective, by being sensitive, discrete but also fair, allowing for extenuating circumstances to be applied when required. An example of this dichotomy is when a student, due to particular circumstances, breaks deadlines. Is a medical certificate always appropriate at times of personal crisis for example? In decisions such as these, intention, practice and fairness could all be fiercely debated within an academic community (Macfarlane, 2004).

It is recognised that more mentors should be instructed to teach their mentees about ethical mentoring and that training programmes should be included within clinical training environments, to ensure ethical mentoring and enhance mentor effectiveness ( Rowe-Johnson, 2018). Interestingly, a study by Lambe in 2010 described little consensus on the agreed generic attributes, qualities and behaviours of a good doctor ( Lambe P and Bristow, 2010). The GMC set out some guiding principles for Admission of MS in 2006:

*‘.recognition that patient care is the primary concern of a doctor. Honesty, empathy, integrity, and an ability to recognise one’s own limitations and those of others .. good communication and listening skills, an ability to make decisions under pressure and remain calm .. to cope with stress and have an understanding of professional issues such as teamwork and respect the contribution of other professions .. Curiosity, creativity, initiative, flexibility and leadership’* ( Medical Schools Council, 2006).

Despite the above declaration about honesty, empathy and integrity, the concept of probity was somewhat controversial in the discussion in Lambe’s publication as it was considered that a doctor might not always be completely honest when acting as the patient’s advocate with other agencies ( Lambe and Bristow, 2010).

*“When you are able to maintain your own highest standards of integrity - regardless of what others may do - you are destined for greatness.”* **-** Napoleon Hill

In a recent experiment investigating ‘Honesty Biases Trustworthiness Impressions’, it was reported that honest individuals were repaid for their honesty with higher trust in a subsequent interaction ( Bellucci and Park, 2020).

Truth, integrity and trust are closely related, indeed truth is essential in learning partnerships, as truth is harmonious with fact and reality but is not always equivalent to perception. Misperceptions sometimes propagate mistruths. Political opinion is an example of how perception can colour the truth or encourage the spread of conspiracy theories ( Weeden and Kurzban, 2014).

*“Veritas est adaequatio rei et intellectus”,(Truth is the adequation of things and intellect)*, Thomas Aquinas (1275-1274)

Integrity is a property that fosters good will and trust. It is an important component of leadership and is also related to credibility and capability.

## Integrity

*Integrity is doing the right thing even when no one is watching.* CS Lewis

Boundaries of academic integrity are not well defined in the collegiate practice of university communities ( McCabe, 2009). The obvious existing rules in academic integrity and ethics, involve absolute transparency, refraining from cheating by falsifying data, plagiarism, and by always disclosing conflicts of interest (McCabe, 2018).

Lecturers with different character traits have expressed dualistic attitudes to dealing with ethical student problems between being objective, by following strictly to the university rules or subjective, by being sensitive, discrete but also fair, allowing for extenuating circumstances to be applied when required. An example of this dichotomy is when a student, due to particular circumstances, breaks deadlines. Is a medical certificate always appropriate at times of personal crisis for example? In decisions such as these, intention, practice and fairness could all be fiercely debated within an academic community (Macfarlane, 2004).

It is recognised that more mentors should be instructed to teach their mentees about ethical mentoring and that training programmes should be included within clinical training environments, to ensure ethical mentoring and enhance mentor effectiveness ( Rowe-Johnson, 2018). Interestingly, a study by Lambe in 2010 described little consensus on the agreed generic attributes, qualities and behaviours of a good doctor ( Lambe and Bristow, 2010). The GMC set out some guiding principles for Admission of MS in 2006-

*‘.recognition that patient care is the primary concern of a doctor. Honesty, empathy, integrity, and an ability to recognise one’s own limitations and those of others .. good communication and listening skills, an ability to make decisions under pressure and remain calm .. to cope with stress and have an understanding of professional issues such as teamwork and respect the contribution of other professions .. Curiosity, creativity, initiative, flexibility and leadership’* ( Medical Schools Council, 2006).

Despite the above declaration about honesty, empathy and integrity, the concept of probity was somewhat controversial in the discussion in Lambe’s publication as it was considered that a doctor might not always be completely honest when acting as the patient’s advocate with other agencies ( Lambe and Bristow, 2010).

*“When you are able to maintain your own highest standards of integrity - regardless of what others may do - you are destined for greatness.”* **-** Napoleon Hill

In a recent experiment investigating ‘Honesty Biases Trustworthiness Impressions’, it was reported that honest individuals were repaid for their honesty with higher trust in a subsequent interaction ( Bellucci and Park, 2020).

Truth, integrity and trust are closely related, indeed truth is essential in learning partnerships, as truth is harmonious with fact and reality but is not always equivalent to perception. Misperceptions sometimes propagate mistruths. Political opinion is an example of how perception can colour the truth or encourage the spread of conspiracy theories ( Weeden and Kurzban, 2014).

*“Veritas est adaequatio rei et intellectus”,(Truth is the adequation of things and intellect)*, Thomas Aquinas (1275-1274)

Integrity is a property that fosters good will and trust. It is an important component of leadership and is also related to credibility and capability.

## Competence and credibility

The reputation of a mentor is also a key factor that may positively impact the level of trust experienced and expressed by the student. A significant indicator that influences the trust relationship between professor and student is the capability of either, which provides an indication of judgement and perceived influence. Recognising capability and potential in the student is also an important factor to build mutual trust. Qualifications, knowledge and experience are strong indicators of the professor’s competence. Academic achievements of the mentor are also evidenced by peer reviewed publications in well renowned journals, presentations, as well as evidence of excellent team work and communication skills; all elements that give a student confidence in the ability of the lecturer and that enhances the TLR. Training and qualifications in teaching and learning enables the lecturer to understand how the student learns and equips them with key pedagogical competencies ( Houldsworth, 2016). Profound understanding of the specialist subject matter in medical science is also important to promote student confidence in their tutor.

The competency of the lecturer has been directly associated with positive student satisfaction in a study that measured the impact of competencies such as subject knowledge, clarity of presentation, interaction with students, teaching creativity, clarifying learning outcome, class activity and lecture notes (Sang *et al.*, 2013).

Excellent skills and training in facilitation of student sessions, using challenging yet probing questions, allows the honest student voice to be heard and often encourages freethinking, motivation, curiosity and creativity thus, a delicate journey for the professor to navigate with the student, requiring careful scaffolded structures in order to accomplish this effectively ( mystudentvoices.com).

Knowledge and skills in course delivery, dedication and attitude to the role of the teacher are also valuable. One study in Chinese higher education discovered that lecturer commitment to students’ academic achievement and lecturer commitment to the social integration of students is both positively related to student satisfaction ( Xiao and Wilkins, 2015).

As well as dedication, accountability must not be forgotten in the subject of trust to avoid the danger of blind trust in the relationship. The basis of trust must be verified as credible, as described by Dean Fink, the leading Canadian educationalist in his book ‘Trust and Verify’. It appears that ‘high trust’ countries produce higher student achievements than low trust countries. Fink describes a spectrum of levels of trust from blind trust to mistrust. Blind trust, when not appropriate, can deviate the direction of the agreed goal ( Fink, 2016).

*‘It is equally an error to trust all men or no man’*-Anon, Latin proverb

It must be recognised that there is an element of risk in entering into these highly valuable trusting relationships and the concept of ‘smart Trust’ where judgement operates as a capability, that enables one to be more discerning about TLRs. This risk landscape of trust needs to be carefully managed. Analysis of the relationship enables the partnership to be developed with wisdom. Occasionally the power differential in the learning relationship can result in coercive behaviours with inappropriate intimacy or closeness where the student may feel uncomfortable, discriminated against or feel too intimidated to complain. This may involve favouritism or discrimination when healthy boundaries are crossed ( Plaut and Baker, 2011).

Predicting and addressing risk when building trust relationships as a tutor for medical students can be structured with these questions about risk and building trust relationships when tutoring medical students?

• What strategies and models of building trusting relationships are used?
• How to continue to build the trust?
• What method is having the greatest impact?
• How to keep the tutoring methods current?
• What impact do these methods make?
• What perceived risk may occur in the future through the current methods?
• Will encountering risk through the changes made cause any disruption to yourself or the students under your care?

The level of transparency and personal information shared is also part of this discernment as it would be unprofessional for a professor to unburden themself of all their health and personal problems to all their students. Thus, competence in discerning the appropriate level of exchange and quality of trust is required ( Covey, 2012).

*Civility has two parts, generosity when it is costly and trust, even when there is risk.’* Stephen Carter

As an example of developing an open and trusting relationship, another personal experience involved the disclosure of mental health issues of some students, which had been kept secret from other previous mentors, in fear of discrimination and exclusion from the course, however once explored, the matter could be supported effectively. Thus some rationality, based on the student evaluation of various factors of character, intent, integrity, and competency, were be taken into account before verifying the trust relationship and enabling them to disclose their personal issues ( Fink, 2016).

Both within academia and beyond medical training, deficient trust relationships often become sour with increased friction, resulting in incompetent behaviour. There may be good intentions but this may be tainted by poor communication. Unless transparency predominates in the relationship, hidden agendas involving politics may result in interpersonal conflict. When there are rivalries between different departments that may result in defensiveness in attitudes and behaviour as well as personally protective interactions between students, academics and departments ( Covey, 2008).

Trusting professional relationships can be modeled to a medical student by the successful collaboration between health care professionals. This is not always such standard practice and the developments of multidisciplinary ideas, with the enhancement of patient-centered care, are the main benefits to these working relationships. Working with other disciplines requires the mutual respect of shared values. In today’s academic climate, collaboration in cooperative complex thinking is an important element in pushing forward the frontiers of research in technology and translational medical science ( Houldsworth, 2019)

Thus trust is not only relevant to TLR but also increases the velocity of research progression by using complimentary concepts, exchanging ideas and interrelating projects that may not have been deemed as complimentary before. Increased trust speeds up the exchange and communication in research, enabling faster progression and decision-making and as such enhances student motivation when applied to academic tutoring.

Cultural competence with sensitivity and awareness is a key learning issue for students and tutors, both as clinicians and educators. Social accountability and cultural awareness in medical training is a fascinating discussion and will complete another publication altogether. This endeavour is life-long, involving self-awareness of assumptions and expectations as much as cultural sensitivity ( Willen, 2013; Abdolmalek *et al*., 2013).

Finally, the impact of capability and competence in teaching is evidenced by the results where appropriate choices of assessment require much attention. Formative assessment drives learning as an external motivator, despite the fact that tutors often strive to promote internal motivation and is also important for programme evaluation and curriculum design ( Preston *et al*., 2020; Hayat *et al*., 2019).

## Intent

The course of action intended differs from competence in that competence is a skill. Transparency, as to the truthful agenda of a mentor, must be reflected upon when entering into the TLR. The sincere desire of a lecturer to facilitate, motivate and inspire an emerging medic, despite the crises and individual trials of each student, can be a dilemma for some academics who celebrate and thrive on consistency and excellence in their students.

Academic staff involved in medical teaching may appear to have limited time and resources, perceived as poor intentions for a student’s development, when their activities compete with the lecturer’s personal research or administrative responsibility. A lack of motivation to facilitate the improved performance of a struggling student, beyond current expectations, may be affected by this. Integrity of intention also applies to the expectations of the lecturer’s role in the university, which may involve extensive periods of research and leave little time for supporting the student. The relationship between teaching and research remains controversial in universities, although expected to be mutually reinforcing, research publication has an important impact on the reputation and ranking of the institution. It seems that current evidence suggests that there are disadvantages to students engaged with staff that are involved in research activities ( Berbegal-Mirabent *et al.,* 2016).

There should be an intention to analyse and diagnose issues through honest reflection, in order to coach the medical student, finding innovative and inspiring solutions that will motivate and energise the student to progress effectively ( Caruso and Rees, 2018).

The teacher’s perception of the relationship is important as it may influence the well being of the teacher or student by influencing the development of the learning relationship. It must be acknowledged that there are positive and negative attracters in such situations. In a positive relationship, with this regard, problematic behaviour from the student may be resolved positively. Negative attractors with low levels of affiliation and non-complimentary views or behaviours are recognised to cause stress and negative emotions. It has been suggested that understanding and managing these kind of scenarios and situations should be implicated in teacher education programmes (Claessens *et al.*, 2016). However, although openness is required within the communion of trust, levels of interaction are not necessarily complimentary with the disclosure of personal information and lecturers should be wise to this.

*‘Positive teacher perceptions of a relationship do not imply equally positive views from the student, nor does a positive perception of the relationship of both parties imply a relationship that is good for student development. One can think for example of a teacher-student relationship that is too intimate and in which a teacher discloses to a student her personal problems; one can argue that such confessions are not beneficial to the students’ learning and school career’* (Luce C.A)

## Neuroscience

**Figure 2.**
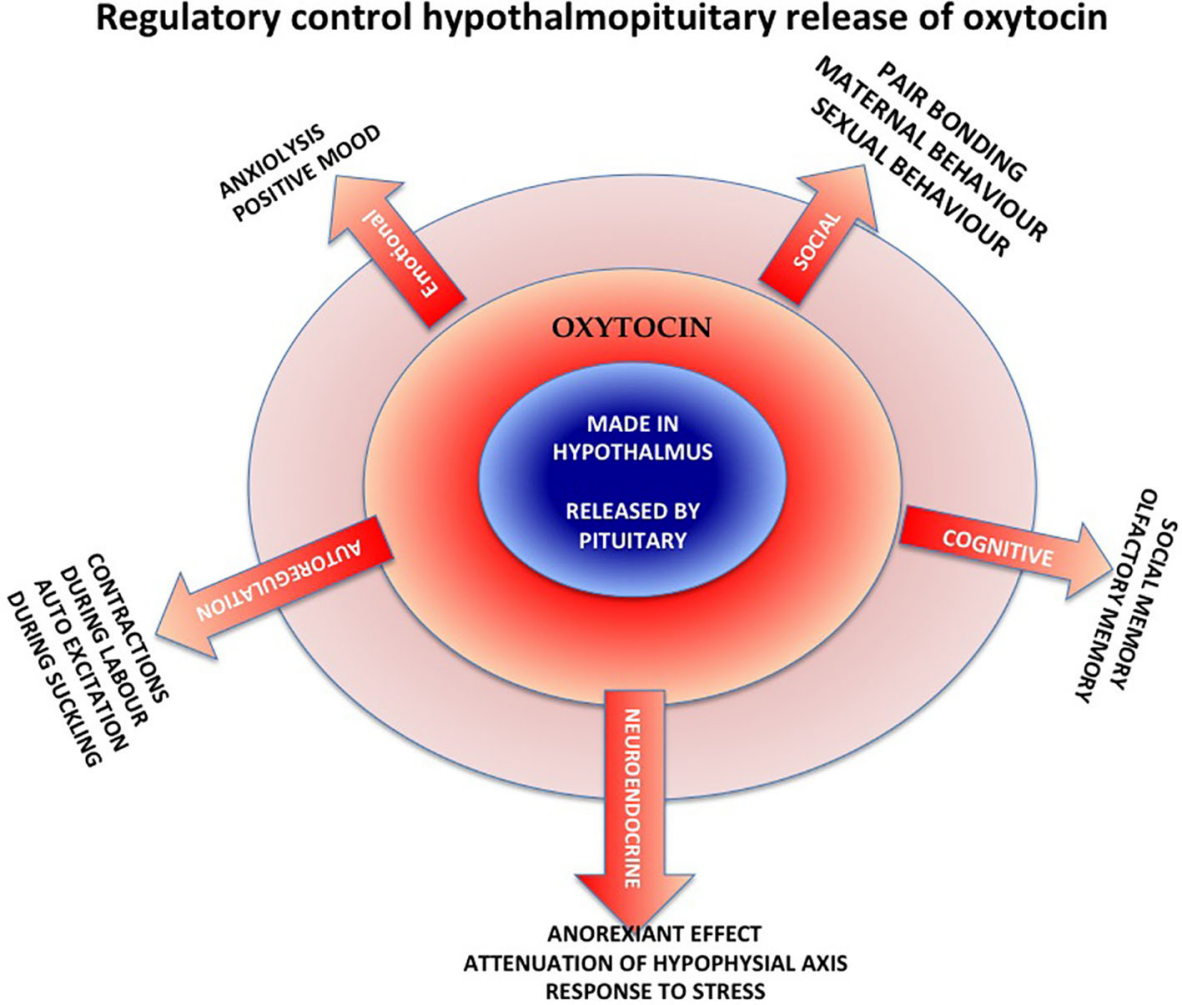
Regulatory Control Releasing Oxytocin

Oxytocin has been described as the ‘moral molecule’ that enhances pair bonding, reciprocity and feel-good factors, also involved in reproduction and childbirth. This neuropeptide is produced in the hypothalamus by oxytocinergic neurons and released by the posterior pituitary ( Figure 2). After childbirth, when suckling stimulates the mother’s nipples, the hormone is released during breastfeeding, causing milk ejection (let down), enhancing bonds with the infant. Oxytocin’s primary function is ‘tend and defend’ ( De Dreu, 2012).

Figure 2. Physiological effects of oxytocin in the human ( Romano *et al*., 2016)

Many aberrant social behaviours, associated with deficiency in oxytocin expression, are recognised, such as, depression, autism and schizophrenia, among several other conditions ( Cochran *et al*., 2013).

Neuroscience recognises that the oxytocin is the brain’s way of transmitting a signal of trust. Prof Paul Zak demonstrated that peripheral oxytocin was the trust hormone and has executed a fascinating set of experiments to prove that oxytocin is the brain’s trust chemical. The level of trust that the participants in the experiment experienced could be predicted by the degree of expression of oxytocin but also how trustworthy they were. By another round of experiments oxytocin was introduced exogenously to the participants of the study, thus replacing the hormone with a synthetic oxytocin, it was demonstrated that oxytocin was the cause of increased levels of trust ( Zak *et al.*, 2004; Zak, 2015).

As with most body-brain axes, there are triggers for the expression or reduction of oxytocin. High stress inhibited the hormone whereas moderate stress, as when solving a problem in a team, enhanced it. The hormone oestrogen enhanced the level of oxytocin whereas testosterone inhibits up to five to ten times more in men ( Zak *et al*., 2004; Zak, 2015; Wu *et al.*, 2020).

In the cross partner nature of this bonding biopsycophysical process, two positive emotions of love and gratitude have been identified. Expressing gratitude and praise greatly enhances relationship satisfaction and fosters relational growth. Perceived partner responsiveness is associated with feeling understood, accepted and cared for and it is suggested that increased oxytocin expression has a role in this relational mechanism. Thus oxytocin and its association with trust and relational relevant behaviours may be a valid factor to consider in the TLR. The association of this mechanism with heterosexual bonding must be a red flag in order to avoid inappropriate TLRs ( Algoe *et al.*, 2017).

After an experiment involving expressions of gratitude between participants CD38 gene is highly expressed in the brain and is associated with oxytocin expression as an important regulator and can be measured in saliva as a measure or a marker of oxytocin expression. There is evidence for a role of the oxytocin system, indexed by genetic variation in CD38, in the social bonding effects of expressed gratitude ( Algoe and Way, 2014; Bonaventura *et al.*, 2016; Jin *et al.*, 2007).

Although attachment theory was initially used to describe children’s relationships in interpersonal neurobiological terms, it has more recently been applied to form an attachment-informed mentorship model that evolves in time and develops trusting relationships focussed on the personal and professional development of the mentee ( Cassidy and Shaver, 1999; Reitz *et al*., 2017).

Perhaps this model could be another aspect in the tutor-training programme.

## Conclusion

There are many different alternative educational strategies in pedagogy/andragogy in medical education establishments, determining alternative curricula including discipline, problem based learning, competency based, organ based, spiral (to name but a few) and discrimination between them, depending on the country of origin or design of curriculum, is common. It seems that excellent doctors emerge from many establishments whether the methodology of medical training is well evaluated and universally approved of or not but the success of the training may be far more dependent on the students’ successful relationships with their teachers and mentors in determining how empowered or motivated they are in that relationship becoming self authors and life long learners with a passion for their craft (Sateesh, 2017).

The increase of trust with time is dependent on several factors discussed in this paper for example, integrity, character, capability and intent, a process that develops from initial introductions to the significant impacts of the relationship. The potential to trust increases with integrity, good character, good intent and professional excellence and speeds the development of the learning relationship engagement ( Figure 1). The risk is in how much disruption these measures create and if they give a competitive edge to the quality of medical training.

The results obtained from excellent learning relationships may be evidenced by assessment and profound understanding of subject matter but perhaps, more importantly, show an improvement in wellbeing, which also may be translated into improved results and internal motivation.

There are many implications for the training of academic tutors discussed above that could enhance current healthcare professional teaching in mentor and coach training, including trust relationships and boundary issues. Further, much more research is required to clarify the many questions that arise from this review.

*‘The rapid pace of disruption brings new risks and threats for organizations. It also requires them to find a different way of managing risk, one that acknowledges the role of trust as a competitive differentiator.’* Amy Brachio

## Take Home Messages

• Trust is an essential component in teacher learner relationships
• Impact of trust on pedagogy, curriculum, delivery, and syllabus content
• Trusting relationships in education instil mutual respect and enhance collaboration determinned by aspects of intention, capability, character, and integrity
• Promotion of independant thinking and self authorship through trust
• Educational theory is supported by neuroscience and psychology

## Notes On Contributors

Dr Houldsworth is an experienced and enthusiastic university lecturer in medical science, a Chartered Scientist, Fellow of the Institute of Biomedical Science, PhD in biomedical science and has interprofessional experience in research and medical education, recently resigned as Assistant Professor at Khalifa University, repatriated to the UK. ORCID iD: https://orcid.org/0000-0003-2692-0537

## Quotations

• Stephen R. Covey
• Brian Tracy
• Anne Frank
• J. T. Duryea
• Abraham Lincoln
• Mahatma Gandhi
• Jack Mezirow
• Clive Staples Lewis
• Medical School Council
• Napoleon Hill
• Thomas Aquinas (1275-1274)
• Anon, Latin proverb
• Luce Claessens
