# Trust Me I'm a Doctor; the Importance of Trust in Promoting High Performance Learning in Medical Education

**DOI:** 10.15694/mep.2020.000184.2

**Published:** 2021-09-29

**Authors:** Annwyne Houldsworth

**Affiliations:** 1 Khalifa University; 2 Khalifa University

**Keywords:** Trust, educational leadership, teaching and learning, professionalism, educational neuroscience, self authorship, mentoring and coaching

## Abstract

This article was migrated. The article was marked as recommended.

This narrative review of the academic literature explores the concept of trust as an essential component of meaningful learning relationships and high performance learning, by establishing definitions and associated factors. These concepts are supported by current research findings about trust relationships in medical education, sourced from Google, MedEdPublish and PubMed Internet searches.

Trust is an essential component of developing bonds and, in particular, the relationship between teacher and learner. The cumulative effects of pedagogy, curriculum, content, and delivery in the teaching and learning of medical students (MS) are well established. The importance of the trust relationship between student and teachers is not widely documented and the current literature is reviewed, addressing aspects of intention, capability, character, and integrity as well as physiological factors, genetic aspects, hormones and neuroscience. Such a detailed dissection and creative analysis of these aspects of medical education, concerning teacher/learner relationships, has not been previously presented.

Trust is often perceived as a soft quality, with respect to education; however, it actually provides an environment of hope and inspirational optimism, where teachers and learners can be authentic about their ‘best selves’. Developing a good character with high emotional intelligence (EI), having transparent intentions in their relationships requires honest reflection and is a key to enhanced integrity.

Building trusting relationships in education instils mutual respect, enhances collaboration, and promotes independent thinking, influenced by transparent and kind mutual interactions. Loyalty and commitment to values and goals ensures the success of the learning environment.

Further, neuroscience, involving psychology experiments demonstrate recent evidence to support the importance of trust in relationships and are considered to be relevant to teaching and learning. Thus, expression of hormones and brain function, associated with trusting relationships and interpersonal bonding is explored.

## Introduction

*“When the trust account is high, communication is easy, instant, and effective.”* -Stephen R. Covey

This narrative review of literature, about trust as an essential component of meaningful learning relationships, defines and validates the nature and elements of trust, establishing and describing the tools and skills required for building successful learning relationships with medical students (MS). Trust provides safety, support, and comfort for both MS and for the patients that they will treat. It is also an important factor between teacher with teacher as well as researcher with researcher and between other professionals. The building of trust, in an educational relationship, is a process that requires key concepts to support its construction. Developing trust involves a number of different factors for the toolkit, some of which are identified and described in this review. Other sources of information, from general education and management settings and from different professional groups, translate well as concepts into medical training, are described and presented, supported by the current literature and data on this subject.

The different sections of the paper were developed from concepts derived from personal professional reflection and training with findings from other research sources. Several educational or management ideas, were the basis of the literature search structure and strategy. Many translational elements were identified from other areas of education and management and resonated with my own reflections and experience as a successful teacher of MS, in a lateral way. In order to determine factors and to support definitions of trust and the learning environment, significant elements were searched on the Internet, using Google, MedEdPublish and PubMed search engines. Several key concepts were identified and investigated, that had commonality with my personal professional experience.

Exploring definitions of trust, Fides is the mythological Roman Goddess of trust, a religious deity with a symbolic narrative, based on an important virtue required for honour and credibility in all contractual arrangements, including marriage. She oversaw the moral integrity of the Romans. From the name of this goddess we derive the word fidelity as a quality of faithfulness or loyalty. The Latin *fidere* means ‘to trust’. Pistis was the Greek equivalent, a spirit associate of truth, trust, honesty and good faith and was a good spirit or personified concept that escaped from Pandora’s box (Thurston, 1898) ( mythencyclopedia.com).

*“The glue that holds all relationships together-including the relationship between the leader and the led-is trust, and trust is based on integrity.”* -Brian Tracy

The concept of trust exceeds the nature of a relationship of alliance and is described as the glue that holds together partnerships. In medicine there are therapeutic alliances between clinician and patient; in law, strategic alliances (agreement between two parties) all of which benefit from the experience of mutual trust (Covey, 2006). Abu Bakr Muhammad ibn Zakariya Al Razi, the freethinking Islamic clinician (936-1013) emphasized the importance of developing trust between patient and doctor ( Lakhtakia, 2014).

**Figure 1.**
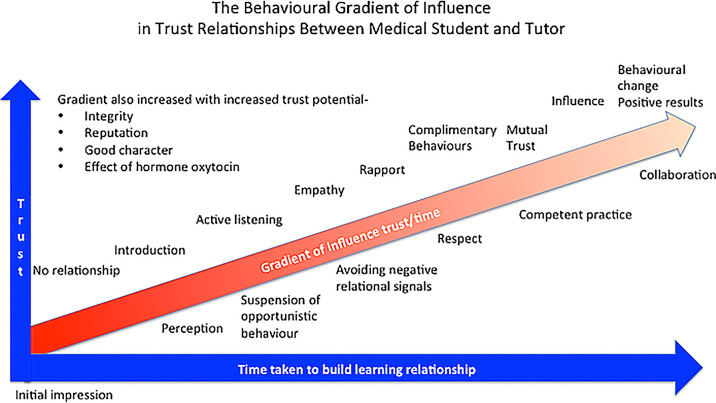
Describing factors that influence the development of trust in a relationship between tutor and medical student. Based on The Behavioural Stairway Influence Mode ( Ireland and Vecchi, 2009).

Trusting teacher learner relationships (TLRs) in education instil mutual respect, enhance collaboration, and promote the independent thinking that results from transparent and kind mutual interactions, also essential skills for the clinician. Indeed, loyalty and commitment to values and goals ensures the success of the learning environment. Many factors enhance trust in the learning environment, adding greater potential to the learning and speed to development ( Figure 1).

Evidence is building in medical education to support the power of mentoring and coaching as a method to improve medical training and education, where high levels of trust are required ( Lovell, 2018).

Often perceived as a soft quality, with respect to education, trust actually provides an environment of hope and inspirational optimism. In such an environment teachers and learners can be authentic about their ‘best selves’, developing good character, where honest reflection is the key to enhanced integrity with transparent intentions in their relationships ( Wilt, Thomas and McAdams, 2019).

Trust is an essential component of the relationship between teacher and learner, particularly as an academic tutor. The cumulative effects of pedagogy, curriculum, content, and delivery in teaching and learning of medical students (MS) are well established and the importance of the relationship between student and learners with particular reference to the concept of trust is explored in a paper by Keiler ( Keiler, 2018). Thus, the moral compass of the participants, involving their individual characters, their integrity, level of competence and credibility as well as their reflective skills, underpinned by physiological factors determining their phenotype, including genes, hormones and neurology, can influence such learning relationships that are explored in this publication.

## Honest Reflection and Evaluation

*‘Deal honestly and objectively with yourself; intellectual honesty and personal courage are the hallmarks of great character.’* -Brian Tracy

A recent study by Bellucci has observed that individuals prefer to put their trust in others who are deemed honest, when they are required to share honest information. An honest reputation influences learning processes for trust-based and social learning interactions. Indeed, honesty is central to trust and trustworthiness ( Bellucci and Park, 2020). With these values in mind, Albert S Humphrey is credited with first developing a strategic planning method to improve performance for evaluation purposes, involving a high degree of honesty in the process where the strengths, weaknesses, opportunities and threats (SWOT) are analysed in a mutually trusting environment ( Businessballs.com).

More recently, it is preferred to for participants to replace the word ‘weakness’ with ‘activities that could be improved’, ‘performed better’ or referred to as ‘limitations’. When there is trust between medical educator and medical student, individual personal reflection needs to be honest, in such a way, that it enables the realistic identification of strengths for both student and mentor to build upon and recognises areas of improvement by self and mutual analysis of tasks, actions, behaviours, attitudes and results. The integrity of this process enables inspired progression and optimism, with a significant identification of improvements to be made in future practice. Best practice is affirmed and empowered and below par practice identified and improved. Evaluation, in a trusting environment, is a powerful tool to develop and ensure best practice in a supportive and empowering ethos. When this is not the case and without trust, the evaluation process can be accusing, discriminatory and disempowering ( Covey, 2008).

The Johari Window is an example of a useful model for reflection that facilitates the understanding of the relationship between themselves and others, as part of the toolkit for professional development, both for student and lecturers ( Verklan, 2007).

It is important that evaluation takes place in a ‘safe place’. The word *amanah (*أمانة) in Arabic means trust from the root word, *amina*, which means state of peace, safety and security. This describes well a safe learning environment for a medical student with their tutor (Islamic Dictionary).

Honesty is often recognised as a character defining attribute.

## Character

*‘The final forming of a person’s character is in their own hands’* -Anne Frank

Character and capability are significant elements to promote trust between individuals. The word ‘character’ is derived from the Greek word *charakter (χαρακτήρ)* and referred to engraving a mark on a coin (χαράσσω) but later used to describe a mark that determines one person from another, as used in the Greek version of the New Testament ( StudyLight.org).

There are distinctive moral and mental characteristics in every individual that may have an important influence on relationships, involving disposition, personality and temperament. Clearly, the individual nature of a person is an aggregation of the traits and features that define them. Moral cognition and decision-making are extremely important traits for the emerging medic.

*“His conscience was the strongest element of his nature. His affections were tender and warm. His whole nature was simple and sincere - he was pure, and then was himself. Such a nature was admirably constituted to direct an heroic struggle on the part of a people proud enough to prefer a guide to a leader, a man commissioned to execute the popular will but, as in his case, strong enough to enforce his own.”* -J. T. Duryea

These particular attributes were written about the character of the sixteenth President of the United States, Abraham Lincoln. The traits that are attributed to President Lincoln were honesty, integrity, courage, fortitude and loyalty, which were exhibited in his words and actions. This enabled the president to instil trust in his colleagues and fellow countrymen (Alvy, 2016).

By deliberately defining personal values and integrity through self-reflection, character can be improved thus enhancing our ability to form trusting relationships.

*‘Character is like a tree and reputation like a shadow what we think of it; the tree is the real thing’.* -Abraham Lincoln

Other attributes commonly attributed to a trustworthy character include consistency, compassion, resourcefulness, good networkers and humility. Thus integrity, character, best intentions, and capability are significant elements to promote trust between individuals. Gandhi’s character is recognised for his congruency in what he feels, thinks, says, and does are all the same.

*‘My life is an indivisible whole and all my activities run into each other...my life is my message’* -Mahatma Gandhi

When a student and mentor clash in personalities and behaviours, the dynamics of the relationship must also be considered. Professionalism may be enhanced by a high degree of emotional intelligence (EI), enabling a tutor to cope with the difficulties encountered in conflicting situations.

EI is where an individual is aware of their own and others emotions and is able to manage them, enabling them to build positive and functional relationships. It is also the ability to perceive emotions in others and express proportionate emotion in response thus facilitating an honest perspective whilst recognising and understanding the cause of those emotions. Finally, managing ones own emotions as teacher, while facilitating those of the student, to achieve the identified goal, demonstrates the role of EI in the TLR.

The Accreditation Council for Graduate Medical Education emphasises the aptitude for non-cognitive characteristics and has identified EI as a core competency ( McPhilemy *et al.*, 2020) ( Leech, 2007). As in many other leadership roles, such as that of a tutor, active listening skills are essential in building trust between individuals while forming the TLR.

An example of character building for the MS involves their role as teachers, mentors and facilitators, developing good facilitation skills, empathy and higher levels of communication. Some medical schools practice the policy of vertical transmission of knowledge where senior students mentor less senior MSs, a situation where ethical mentor ability is required. As a personal observation, junior MSs often have a great deal of trust in their high achieving senior MSs but the quality of the transferred knowledge must be verified for accuracy as some information may be misinterpreted by students. The more recent ‘student as teacher’ programmes equip students with strategies helping MS to be competent teachers (Jasmine and Taylor, 2017).

*“to negotiate and act on our own purposes, values, feelings, and meanings rather than those we have uncritically assimilated from others”* (Mezirow, 2000)

The above quotation from Mezirow describes transformative learning, using honest reflection, questioning and critical thinking to challenge underlying assumptions and beliefs, being more inclusive, open and discriminating. Blind trust is when the knowledge or concepts presented to the student are completely accepted, whereas the process of self-authorship undergoes critical analysis of those accepted assumptions, gaining a new internal perspective or authority, a process also related to developing good character.

There is an increasing interest in Robert Kegan’s conceptual theory of self-authorship within a medical education context ( Kegan, 1994). Individuals are not born with self-authorship but they become independent of external values and loyalties. Students achieve personal authority through a process involving the three-dimensional elements of cognitive, interpersonal and intrapersonal development. The student gains the ability to interpret experiences, based on a well-developed inner voice and not always on the values of other people. They learn to trust their own internal voice. In other words the medical student begins to be discerning and strong enough in character to stand apart from the mainstream and discern whom they should trust. The student takes responsibility for their own learning and is no longer dependent on external forces but becomes a life long self-directed learner ( Sandars and Jackson, 2015).

Enabling this personal development of character to take place involves changing and evaluating the curricula, putting an emphasis on teacher facilitation, where students can attain their true potential. An honest reflection on the constraining factors that inhibit the students to attain their true aspirations is required to alter the curricula delivery. This is exemplified in the ‘capability approach’ to medical education, where the teaching is student-centred and not lecturer-centred. Not forgetting that a medical student’s learning should also be patient-centred and case study narratives enable this connection with medical science theory ( Sandars and Sarojini, 2015). Personal use of patient narrative, as a pedagogic tool during medical school teaching of basic science, involving creative reflection, has proved extremely successful, well received by students, and is reported to improve the student’s understanding and compassion of patient experiences ( Milota *et al*., 2019).

Trust remains a significant factor in dealing with student problems and the encouragement of honest recognition of feelings expressed or repressed, along with understanding the cause of those emotions can enable a student to achieve their true potential ( Valiente, Swanson and Eisenberg, 2012).

Integrity and character are often misjudged as being interrelated but actually integrity is about being uncompromisingly honest whereas character is about consistency and the distinctive nature of an individual ( Oxford English Dictionary).

## Integrity

*‘Integrity is doing the right thing even when no one is watching.’* -CS Lewis

Boundaries of academic integrity are not well defined in the collegiate practice of university communities ( McCabe, 2009). The obvious existing rules in academic integrity and ethics, involve absolute transparency, refraining from cheating by falsifying data, plagiarism, and by always disclosing conflicts of interest (McCabe, 2018).

Lecturers possessing different character traits have expressed dualistic attitudes to dealing with ethical student problems, between being objective, by following strictly to the university rules, or subjective by being sensitive, discrete but also fair, allowing for extenuating circumstances to be applied when required. An example of this dichotomy is when a student, due to particular circumstances, breaks deadlines. Is a medical certificate always appropriate at times of personal crisis, for example? In decisions, such as these, intention, practice and fairness could all be fiercely debated within an academic community (Macfarlane, 2004).

It is recognised that more mentors should be instructed to teach their mentees about ethical mentoring and that training programmes should be included within clinical training environments, to ensure ethical mentoring and to enhance mentor effectiveness ( Rowe-Johnson, 2018). Interestingly, a study by Lambe in 2010 described little consensus on the agreed generic attributes, qualities and behaviours of a good doctor ( Lambe and Bristow, 2010). The GMC set out some guiding principles for Admission of MS in 2006-

*‘..recognition that patient care is the primary concern of a doctor. Honesty, empathy, integrity, and an ability to recognise one’s own limitations and those of others ... good communication and listening skills, an ability to make decisions under pressure and remain calm ... to cope with stress and have an understanding of professional issues such as teamwork and respect the contribution of other professions ... Curiosity, creativity, initiative, flexibility and leadership’* ( Medical Schools Council, 2006) *.*

Despite the above declaration about honesty, empathy and integrity, the concept of probity was somewhat controversial in the discussion in Lambe’s publication as it was considered that a doctor might not always be completely honest when acting as the patient’s advocate with other agencies ( Lambe and Bristow, 2010).

*‘When you are able to maintain your own highest standards of integrity - regardless of what others may do - you are destined for greatness.*’-Napoleon Hill

In a recent experiment investigating ‘Honesty Biases Trustworthiness Impressions’, it was reported that honest individuals were repaid for their honesty with higher trust in a subsequent interaction ( Bellucci and Park, 2020).

Truth, integrity and trust are closely related, indeed truth is essential in learning partnerships, as truth is harmonious with fact and reality but is not always equivalent to perception. Misperceptions sometimes propagate mistruths. Political opinion is an example of how perception can colour the truth or encourage the spread of conspiracy theories ( Weeden and Kurzban, 2014).

*‘Veritas est adaequatio rei et intellectus’* *(Truth is the adequation of things and intellect)* -Thomas Aquinas (1275-1274)

Integrity is a property that fosters good will and trust. It is an important component of leadership and is also related to credibility and capability.

## Competence and Credibility

The reputation of a mentor is also a key factor that may positively impact the level of trust experienced and expressed by the student. A significant indicator that influences the trust relationship between professor and student is the capability of either, which provides an indication of judgement and perceived influence. Recognising capability and potential in the student is also an important factor to build mutual trust. Qualifications, knowledge and experience are strong indicators of the professor’s competence. Academic achievements of the mentor are also evidenced by peer reviewed publications in well renowned journals, presentations, as well as evidence of excellent team work and communication skills; all elements that give a student confidence in the ability of the lecturer and that enhances the TLR. Training and qualifications in teaching and learning enables the lecturer to understand how the student learns and equips them with key pedagogical competencies ( Houldsworth, 2016). Profound understanding of the specialist subject matter in medical science is also important to promote student confidence in their tutor.

The competency of the lecturer has been directly associated with positive student satisfaction in a study that measured the impact of competencies such as subject knowledge, clarity of presentation, interaction with students, teaching creativity, clarifying learning outcome, class activity and lecture notes (Sang *et al.*, 2013).

Excellent skills and training in facilitation of student sessions, using challenging yet probing questions, allows the honest student voice to be heard and often encourages freethinking, motivation, curiosity and creativity thus, a delicate journey for the professor to navigate with the student, requiring careful scaffolded structures in order to accomplish this effectively ( mystudentvoices.com).

Knowledge and skills in course delivery, dedication and attitude to the role of the teacher are also valuable. One study in Chinese higher education discovered that lecturer commitment to students’ academic achievement and lecturer commitment to the social integration of students is both positively related to student satisfaction ( Xiao and Wilkins, 2015).

As well as dedication, accountability must not be forgotten in the subject of trust to avoid the danger of blind trust in the relationship. The basis of trust must be verified as credible, as described by Dean Fink, the leading Canadian educationalist in his book ‘Trust and Verify’. It appears that ‘high trust’ countries produce higher student achievements than low trust countries. Fink describes a spectrum of levels of trust from blind trust to mistrust. Blind trust, when not appropriate, can deviate the direction of the agreed goal ( Fink, 2016).

*‘It is equally an error to trust all men or no man’* -Anon, Latin proverb

It must be recognised that there is an element of risk in entering into these highly valuable trusting relationships and the concept of ‘Smart Trust’ where judgement operates as a capability, that enables one to be more discerning about TLRs. This risk landscape of trust needs to be carefully managed. Analysis of the relationship enables the partnership to be developed with wisdom. Occasionally the power differential in the learning relationship can result in coercive behaviours with inappropriate intimacy or closeness where the student may feel uncomfortable, discriminated against or feel too intimidated to complain. This may involve favouritism or discrimination when healthy boundaries are crossed ( Plaut and Baker, 2011).

Predicting and addressing risk when building trust relationships as a tutor for medical students can be structured with these questions about risk and building trust relationships when tutoring medical students?

• What strategies and models of building trusting relationships are used?
• How to continue to build the trust?
• What method is having the greatest impact?
• How to keep the tutoring methods current?
• What impact do these methods make?
• What perceived risk may occur in the future through the current methods?
• Will encountering risk through the changes made cause any disruption to yourself or the students under your care?

The level of transparency and personal information shared is also part of this discernment as it would be unprofessional for a professor to unburden themself of all their health and personal problems to all their students. Thus, competence in discerning the appropriate level of exchange and quality of trust is required ( Covey, 2012).

*‘Civility has two parts, generosity when it is costly and trust, even when there is risk.’* -Stephen Carter

As an example of developing an open and trusting relationship, another personal experience involved the disclosure of mental health issues of some students, which had been kept secret from other previous mentors, in fear of discrimination and exclusion from the course, however once explored, the matter could be supported effectively. Thus some rationality, based on the student evaluation of various factors of character, intent, integrity, and competency, were be taken into account before verifying the trust relationship and enabling them to disclose their personal issues ( Fink, 2016).

Both within academia and beyond medical training, deficient trust relationships often become sour with increased friction, resulting in incompetent behaviour. There may be good intentions but this may be tainted by poor communication. Unless transparency predominates in the relationship, hidden agendas involving politics may result in interpersonal conflict. When there are rivalries between different departments that may result in defensiveness in attitudes and behaviour as well as personally protective interactions between students, academics and departments ( Covey, 2008).

Trusting professional relationships can be modeled to a medical student by the successful collaboration between health care professionals. This is not always such standard practice and the developments of multidisciplinary ideas, with the enhancement of patient-centered care, are the main benefits to these working relationships. Working with other disciplines requires the mutual respect of shared values. In today’s academic climate, collaboration in cooperative complex thinking is an important element in pushing forward the frontiers of research in technology and translational medical science ( Houldsworth, 2019).

Thus trust is not only relevant to TLR but also increases the velocity of research progression by using complimentary concepts, exchanging ideas and interrelating projects that may not have been deemed as complimentary before. Increased trust speeds up the exchange and communication in research, enabling faster progression and decision-making and as such enhances student motivation when applied to academic tutoring.

Cultural competence with sensitivity and awareness is a key learning issue for students and tutors, both as clinicians and educators. Social accountability and cultural awareness in medical training is a fascinating discussion and will complete another publication altogether. This endeavour is life-long, involving self-awareness of assumptions and expectations as much as cultural sensitivity ( Willen, 2013) (Abdolmalek *et al*., 2013).

Finally, the impact of capability and competence in teaching is evidenced by the results where appropriate choices of assessment require much attention. Formative assessment drives learning as an external motivator, despite the fact that tutors often strive to promote internal motivation and is also important for programme evaluation and curriculum design ( Preston *et al*., 2020) ( Hayat *et al*., 2019).

## Intent

The course of action intended differs from competence in that competence is a skill. Transparency, as to the truthful agenda of a mentor, must be reflected upon when entering into the TLR. The sincere desire of a lecturer to facilitate, motivate and inspire an emerging medic, despite the crises and individual trials of each student, can be a dilemma for some academics who celebrate and thrive on consistency and excellence in their students.

Academic staff involved in medical teaching may appear to have limited time and resources, perceived as poor intentions for a student’s development, when their activities compete with the lecturer’s personal research or administrative responsibility. A lack of motivation to facilitate the improved performance of a struggling student, beyond current expectations, may be affected by this. Integrity of intention also applies to the expectations of the lecturer’s role in the university, which may involve extensive periods of research and leave little time for supporting the student. The relationship between teaching and research remains controversial in universities, although expected to be mutually reinforcing, research publication has an important impact on the reputation and ranking of the institution. It seems that current evidence suggests that there are disadvantages to students engaged with staff that are involved in research activities ( Berbegal-Mirabent *et al.,* 2016).

There should be an intention to analyse and diagnose issues through honest reflection, in order to coach the medical student, finding innovative and inspiring solutions that will motivate and energise the student to progress effectively ( Caruso and Rees, 2018).

The teacher’s perception of the relationship is important as it may influence the well being of the teacher or student by influencing the development of the learning relationship. It must be acknowledged that there are positive and negative attracters in such situations. In a positive relationship, with this regard, problematic behaviour from the student may be resolved positively. Negative attractors with low levels of affiliation and non-complimentary views or behaviours are recognised to cause stress and negative emotions. It has been suggested that understanding and managing these kind of scenarios and situations should be implicated in teacher education programmes (Claessens *et al.*, 2016). However, although openness is required within the communion of trust, levels of interaction are not necessarily complimentary with the disclosure of personal information and lecturers should be wise to this.

*‘Positive teacher perceptions of a relationship do not imply equally positive views from the student, nor does a positive perception of the relationship of both parties imply a relationship that is good for student development. One can think for example of a teacher-student relationship that is too intimate and in which a teacher discloses to a student her personal problems; one can argue that such confessions are not beneficial to the students’ learning and school career’* (Luce C. A)

## Neuroscience

*‘Neuroscience is by far the most exciting branch of science because the brain is the most fascinating object in the universe. Every human brain is different- the brain makes each human unique and defines who he or she is.*’-Stanley B. Prosiner

Finally, educational theory is increasingly influenced by concepts of neuroscience and it’s impact on learning. Concepts of neuroscience involve some factors that affect relationship and psychology.

**Figure 2.**
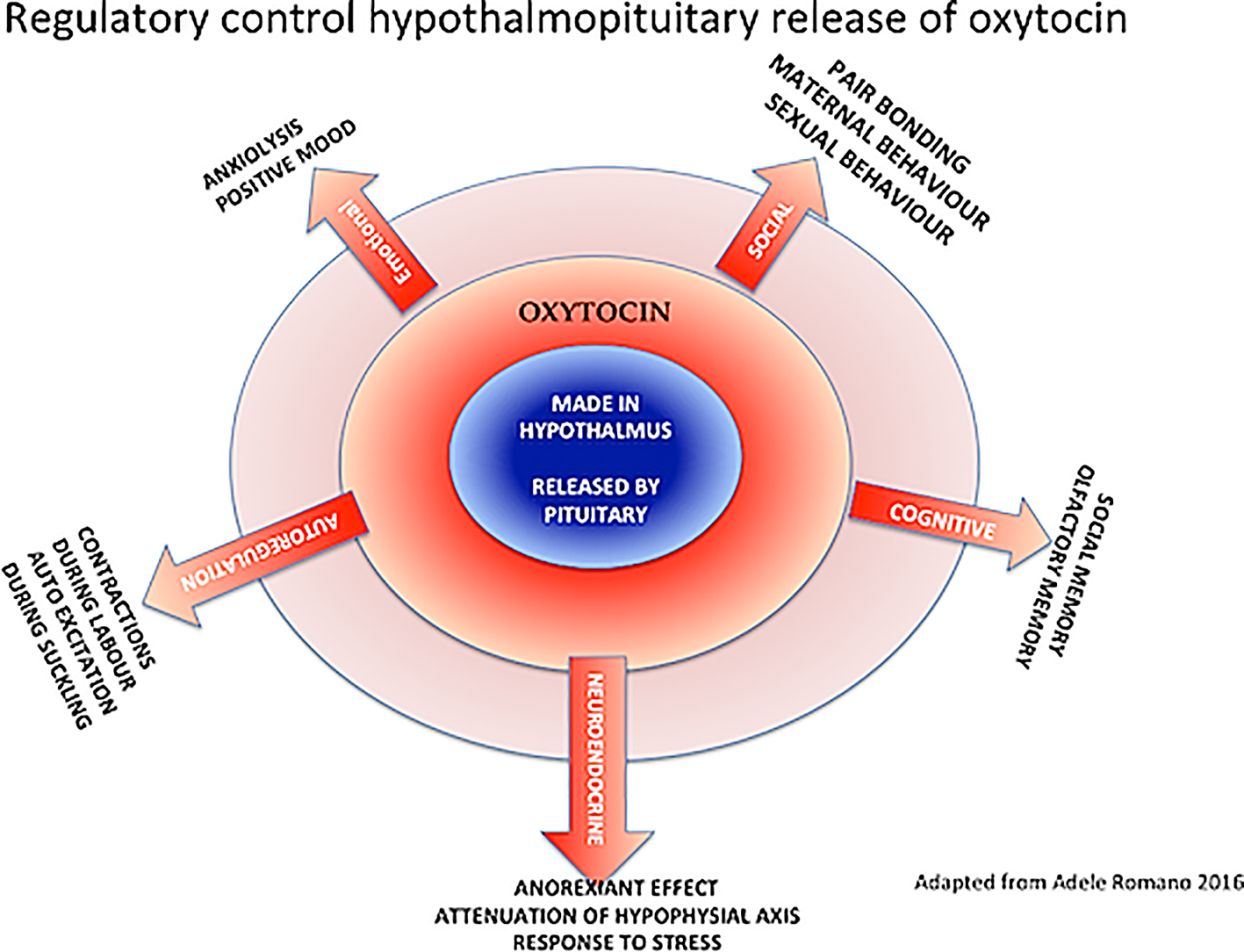
Physiological effects of oxytocin in the human ( Romano *et al*., 2016)

With this in mind, oxytocin has been described as the ‘moral molecule’ that enhances pair bonding, reciprocity and feel-good factors, also involved in reproduction and childbirth. This neuropeptide is produced in the hypothalamus by oxytocinergic neurons and released by the posterior pituitary ( Figure 2). After childbirth, when suckling stimulates the mother’s nipples, the hormone is released during breastfeeding, causing milk ejection (let down), enhancing bonds with the infant. Oxytocin’s primary function is ‘tend and defend’ ( De Dreu, 2012).

Many aberrant social behaviours, associated with deficiency in oxytocin expression, are recognised, such as, depression, autism and schizophrenia, among several other conditions ( Cochran *et al*., 2013).

Neuroscience recognises that the oxytocin is the brain’s way of transmitting a signal of trust. Prof Paul Zak demonstrated that peripheral oxytocin was the trust hormone and has executed a fascinating set of experiments to prove that oxytocin is the brain’s ‘trust chemical’. The level of trust that the participants in the experiment experienced could be predicted by the degree of expression of oxytocin but also how trustworthy they were. By another round of experiments oxytocin was introduced exogenously to the participants of the study, thus replacing the hormone with a synthetic oxytocin, it was demonstrated that oxytocin was the cause of increased levels of trust ( Zak, 2004, 2015).

As with most body-brain axes, there are triggers for the expression or reduction of oxytocin. High stress inhibited the hormone whereas moderate stress, as when solving a problem in a team, enhanced it. The hormone oestrogen enhanced the level of oxytocin whereas testosterone inhibits up to five to ten times more in men ( Zak, 2015)( Zak *et al.*, 2004)( Wu *et al.*, 2020).

In the cross partner nature of this bonding biopsycophysical process, two positive emotions of love and gratitude have been identified. Expressing gratitude and praise greatly enhances relationship satisfaction and fosters relational growth. Perceived partner responsiveness is associated with feeling understood, accepted and cared for and it is suggested that increased oxytocin expression has a role in this relational mechanism. Thus oxytocin and its association with trust and relational relevant behaviours may be a valid factor to consider in the TLR. The association of this mechanism with heterosexual bonding must be a red flag in order to avoid inappropriate TLRs ( Algoe *et al.*, 2017).

After an experiment involving expressions of gratitude between participants CD38 gene is highly expressed in the brain and is associated with oxytocin expression as an important regulator and can be measured in saliva as a measure or a marker of oxytocin expression. There is evidence for a role of the oxytocin system, indexed by genetic variation in CD38, in the social bonding effects of expressed gratitude ( Algoe and Way, 2014) (Bonaventura *et al.*, 2016) (Jin *et al.*, 2007).

Although attachment theory was initially used to describe children’s relationships in interpersonal neurobiological terms, it has more recently been applied to form an attachment-informed mentorship model that evolves in time and develops trusting relationships focussed on the personal and professional development of the mentee ( Cassidy and Shaver, 1999) (Reitz *et al*., 2017).

Perhaps, the attachment theory mentorship model could be another aspect in the tutor-training programme in presenting methods to improve TLRs.

## Methodology

• The process started with personal reflection of successful experiences in learning relationships and some historic qualitative data gathered from conversations with students and staff, during my medical teaching career. Also observation of difficulties and lessons learnt were important.
• Identifying established, historical and cultural examples of building trust relationships and trustworthy personalities.
• Transferable concepts from general education and management theory were explored for common concepts and identified as a useful starting point providing suggestions about trust in a general sense.
• More specifically, concepts of trust from medical educational theory, related to the teacher-learner relationships were also identified and included in the searches.
• Establishing significant factors, such as, integrity, character and intention and how they related to medical education were searched individually.
• Aspects of competence and credibility were also researched in the literature. Other factors investigated were honest reflective practice and evaluation of learning identifying relational outcomes and their contribution to trusting relationships. Some interprofessional papers were read for this purpose.
• Emotional contribution, psychological factors and neuroscience were all identified, as important elements integral to the teacher/learner trust relationship.
• Each of the above identified concepts and subjects were individually researched in Google, MedEdPublish and PubMed search engines, and the results of the literature search were presented and discussed with all relevant publications referenced. 58 publications were included and referenced and 15 relevant quotations both historical and current sources were included.
• No dates were set for accessing publications, in order to maintain both a historical and contemporary perspective on trust.
• Quotations were chosen from related quotation websites and recorded sayings after Google searches, websites included were www.brainyquote.com, www.picturequotes.com, www.best-quotations.com and www.goodreads.com.

## Conclusion

This paper establishes the importance of the increase of trust with time, by reviewing academic literature surrounding the subject. Trust in TLR relationships is dependent on several factors discussed, for example, integrity, character, capability and intent; a process that develops from initial introductions to the significant impacts of the relationship. The potential to trust increases with integrity, good character, good intent and professional excellence and speeds the development of the learning relationship engagement ( Figure 1). The risk is in how much these measures are disrupted, if they give a competitive edge to the quality of medical training.

The results obtained from excellent learning relationships may be evidenced by assessment and profound understanding of subject matter but perhaps, more importantly, show an improvement in wellbeing, which also may be translated into improved results and internal motivation.

There are many implications for the training of academic tutors discussed above that could enhance current healthcare professional teaching in mentor and coach training, including trust relationships and boundary issues.

Further, much more research is required to clarify the many questions that arise from this review.

*‘The rapid pace of disruption brings new risks and threats for organizations. It also requires them to find a different way of managing risk, one that acknowledges the role of trust as a competitive differentiator.’* -Amy Brachio

## Take Home Messages

• Trust is an essential component in teacher learner relationships
• Review of the literature, supporting the importance of trusting relationships in medical education, using Google, MedEdPublish and PubMed seach engines
• Impact of trust on pedagogy, curriculum, delivery, and syllabus content
• Trusting relationships in education instil mutual respect and enhance collaboration determinned by aspects of intention, capability, character, and integrity
• Promotion of independant thinking and self authorship through trust
• Educational theory is supported by neuroscience and psychology

## Notes On Contributors

Dr Houldsworth is an experienced and enthusiastic university lecturer in medical science, a Chartered Scientist, Fellow of the Institute of Biomedical Science, PhD in biomedical science and has interprofessional experience in research and medical education, recently resigned as Assistant Professor at Khalifa University, repatriated to the UK. ORCID iD: https://orcid.org/0000-0003-2692-0537

## Quotations

• Stephen R. Covey
• Brian Tracy
• Anne Frank
• J. T. Duryea
• Abraham Lincoln
• Mahatma Gandhi
• Jack Mezirow
• Clive Staples Lewis
• Medical School Council
• Napoleon Hill
• Thomas Aquinas (1275-1274)
• Anon, Latin proverb
• Luce Claessens
• Stanley B Prosiner
