# Supplementary material for: Trust Me I'm a Doctor; the Importance of Trust in Promoting High Performance Learning in Medical Education
Source: MedEdPublish (2016). 2021 Sep 29;9:184. Originally published 2020 Aug 27. [Version 2] doi: 10.15694/mep.2020.000184.2 (PMC10929691; doi:10.15694/mep.2020.000184.2)
Supplement: Peer review history

# Reviewer report: Reviewer response for version 2

**DOI:** 10.21956/mep.20271.r31467

**Published:** 2021-10-11

**Authors:** Mohamed Al-Eraky

**Affiliations:** 1 University of Dammam

This review has been migrated. The reviewer awarded 3 stars out of 5 Thank you. This is an interesting article. I concur with the findings of my colleague Ken and Gert, who offered valuable insights in their reviews. I would like to add a comment on Figure 1. The gain of trust may not go in a linear fashion. Also, trust it’s a highly contextual construct that contributes to professionalism in different cultures. This point may need further elaboration from authors. Good luck.

Reviewer Expertise:

NA

No decision status is available

# Reviewer report: Reviewer response for version 2

**DOI:** 10.21956/mep.20271.r31465

**Published:** 2021-10-03

**Authors:** Ken Masters

**Affiliations:** 1 Sultan Qaboos University

This review has been migrated. The reviewer awarded 3 stars out of 5 The author has made an effort to correct many of the problems in Version 1, but there are still some issues that were left unaddressed.• Perhaps the most striking is the series of quotations that exist without reference. (Simply giving a quotation followed by a name, even if well known, is not appropriate. A proper and formal reference for every quotation is required.). So, stating names (examples are Tracey, Frank, Hill and Lewis) without a source is a hindrance. And, when they are placed in the middle of the text, without any comment or link to the surrounding text, their insertion into the text appears somewhat arbitrary. In other cases (Covey), there is no source stated, although texts by that author, in conjunction with other authors, are listed, so the source might or might not be listed. (And a listing of the sites consulted at the end of the Methodology is, unfortunately, not a substitute for the correct citation process).• Similarly, it is frequently difficult to distinguish between the author’s opinion and opinion drawn from other literature, primarily because self-standing comments are made. For example, “Integrity is a property that fosters good will and trust. It is an important component of leadership and is also related to credibility and capability.” Is this the author’s opinion, or is it something drawn from the preceding literature? In a literature review, it is essential that this distinction be made (and that the author’s own opinion, unless derived directly and obviously from the literature cited, or commenting directly on the literature, should be kept to a minimum, or not given at all.) This is a literature review, not a personal opinion piece.So, I can see that that the author has made an effort to make the paper more readable, but, as a literature review, its potential contribution to the field has been hampered by practices that require improvement.

Reviewer Expertise:

NA

No decision status is available

# Reviewer report: Reviewer response for version 2

**DOI:** 10.21956/mep.20271.r31466

**Published:** 2021-10-01

**Authors:** Gert Van Zyl

**Affiliations:** 1 University of the Free State

This review has been migrated. The reviewer awarded 5 stars out of 5 What a lovely article. It is true that trust is an essential component of the relationship between teacher and learner, particularly as an academic tutor as indicated in the article. The paper is a good indicating that trust plays in medical education. Well written and a article of quality.

Reviewer Expertise:

NA

No decision status is available

# Reviewer report: Reviewer response for version 1

**DOI:** 10.21956/mep.19006.r27268

**Published:** 2021-02-11

**Authors:** Richard Hays

**Affiliations:** 1 James Cook University

This review has been migrated. The reviewer awarded 2 stars out of 5 I enjoyed reading this article. Trust is obviously important in both patient care and learning and a lot of important information is included. However, I am not sure if it a literature review or, if it is, how it was conceived and structured from what sources. To be a literature review, the article needs a background to define the issue being reviewed, a methods section that explains how the search was conducted and then some kind of synthesis that addresses the original issues. This reads (well) like a kind of potted summary of selected sources around topics of personal interest as well as importance. How were these topics selected? If a revision is contemplated, following a more traditional review format would earn more stars.

Reviewer Expertise:

NA

No decision status is available

# Reviewer report: Reviewer response for version 1

**DOI:** 10.21956/mep.19006.r27267

**Published:** 2020-10-07

**Authors:** Ken Masters

**Affiliations:** 1 Sultan Qaboos University

This review has been migrated. The reviewer awarded 2 stars out of 5 The paper deals with the issue of trust in medical education. While the paper does raise an important issue, it suffers from several challenges, primarily the structure, and these will need to be addressed.• Overall the paper does have a great deal of useful information, but the structure of the paper does not lend itself to easy reading. At the moment, the paper appears to be a listing of a large number of thoughts about the concept of trust, with no real argument or flow. I really think the paper could probably retain almost all of its current content, but needs to be re-shaped. For example, in a paragraph like this:--A recent study by Bellucci has observed that individuals prefer trusting honest others when required to share honest information. An honest reputation influences learning processes for trust-based interactions in social learning social interactions and honesty is central to trust and trustworthiness (Bellucci and Park, 2020). Albert S Humphrey is credited with first developing the strengths, weaknesses, opportunities and threats (SWOT) analysis, a strategic planning method to improve performance, for evaluation purposes--There does not appear to be any logical connection between the first two sentences and the third.• Elsewhere, also, the paper has useful thoughts, but appears to be listing these thoughts without a path. In addition, the paper is sometimes peppered with quotations, but these quotations do not have an obvious connection to the paragraphs that follow. A starting point would be to re-write the Abstract in such a way that the flow of the paper is described, and then it gives the overall direction of the paper.• In this vein, the sudden switch to the Neuroscience is jarring, as the paper changes direction, and the link between this section and the previous, more philosophical view, is not clear, apart from a single sentence at the end of the section (“Perhaps this model could be another aspect in the tutor-training programme.”) This sentence does require further expansion to see how the model could be used.• The information under the heading “Character” and “Integrity” is identical.• The Conclusion appears to introduce new material; it should rather be used to sum up and reflect on what has been said in the body of the paper.Smaller Issues:• There are several areas where the paper could benefit from some extra punctuation, especially commas, to aid the reading. For example, a sentence like “An honest reputation influences learning processes for trust-based interactions in social learning social interactions and honesty is central to trust and trustworthiness” is really confusing. There are quite a few others that have the same problem, so a careful proof-read would be useful.• In the line, “the concept of trust is explored in this paper (Keiler, 2018)” it is difficult to determine whether “this paper” refers to the current paper being read, or Keiler, 2018.• “prefer trusting honest others” I think this is an error, and needs to be corrected.So, I think the paper has the core of some useful insight, but does need to be re-worked into a shape that looks less like a listing of ideas and more like a coherent argument or process of thought. I look forward to Version 2 in which the issues have been addressed.

Reviewer Expertise:

NA

No decision status is available

# Reviewer report: Reviewer response for version 1

**DOI:** 10.21956/mep.19006.r27269

**Published:** 2020-09-03

**Authors:** Richard F Heller

**Affiliations:** 1 People's Open Access Education Initiative (Peoples-uni)

This review has been migrated. The reviewer awarded 5 stars out of 5 This is a very important area, and I congratulate the author on bringing it to our attention. The review highlights the potential importance of trust on student outcomes as well as providing an understanding of its biochemical basis and historical background.

Reviewer Expertise:

NA

No decision status is available
